# Modification of Fluorescent Photoinduced Electron Transfer (PET) Sensors/Switches To Produce Molecular Photo-Ionic Triode Action**

**DOI:** 10.1002/anie.201310939

**Published:** 2014-02-26

**Authors:** Allen J M Huxley, Marc Schroeder, H Q Nimal Gunaratne, A Prasanna de Silva

**Affiliations:** School of Chemistry and Chemical Engineering, Queen's UniversityBelfast BT9 5AG (Northern Ireland)

**Keywords:** electron transfer, fluorescence, molecular devices, molecular switches, triodes

## Abstract

The fluorophore-spacer_1_-receptor_1_-spacer_2_-receptor_2_ system (where receptor_2_ alone is photoredox-inactive) shows ionically tunable proton-induced fluorescence off-on switching, which is reminiscent of thermionic triode behavior. This also represents a new extension to modular switch systems based on photoinduced electron transfer (PET) towards the emulation of analogue electronic devices.

Fluorescent photoinduced electron transfer (PET) sensors/switches[1–4] are a well-established application of molecular devices, to the point of real-life deployment worldwide in blood electrolyte diagnostics.[5–8] Important picosecond laser studies on fluorescent PET sensors/switches have demonstrated the transient existence of radical ion species,[9–11] and thus designers can proceed with confidence. As a result of their modular fluorophore-spacer-receptor construction, fluorescent PET systems are very amenable to modification in terms of the format, as well as in terms of the detailed functionalities. The latter approach has yielded many individual examples of sensors and switches based on fluorescence which target important analytes.[12–15] On the other hand, the former approach has the potential to set up new areas of endeavor and application, which is exploited here.

The controllable quenching of molecular fluorescence[1,2] can be exploited to build switchable systems which emulate familiar electronic devices. Some of these molecular systems have unique applications which are inconvenient for their electronic counterparts, such as wireless operation in micrometric spaces.[16] The first molecular logic gate[17–23] **1** (an advanced molecular switch[24]) was a fluorophore-spacer_1_-receptor_1_-spacer_2_-receptor_2_ system,[25,26] where two photoinduced electron transfer (PET)[4,27] channels arising from the two receptors were controlled by binding H^+^ and Na^+^ ions, respectively, and thus the fluorescence output corresponded to photo-ionic AND logic. Strong fluorescence emerges only when all PET processes are suppressed (Figure 1 a).[4] Related, but distinct, fluorophore-spacer_1_-receptor_1_-spacer_2_-receptor_2_ systems,[28] where both receptors respond to H^+^ ions, for example, **2**, give rise to fluorescent off-on-off action, which can correspond to ternary logic behavior.[22] We now demonstrate aspects of molecular photo-ionic triode action for the first time by structurally mutating the fluorophore-spacer_1_-receptor_1_-spacer_2_-receptor_2_ system **1** into a novel format exemplified by **3**, where the convenient photoredox capability of receptor_2_ is removed from **1** (Figure 1 b). Nearly 20 distinct formats of luminescent PET switching systems, each possessing its own defining features and applications, are known.[26] Fluorescence off-on switching is, therefore, controlled within **3** by selective ion binding of receptor_1_. This switching profile is influenced by the orthogonally selective ion binding of receptor_2_, which is forced into a secondary role (Figure 1 b). The amine receptor_1_ within **3** would bind H^+^ instead of alkali and alkaline earth cations. The crown ether receptor_2_ within **3** would bind alkali and alkaline earth cations instead of H^+^. This photo-ionic triode action complements molecular all-photonic triode behavior, which was reported recently by Gust, Moore, Moore, and co-workers.[29] Molecular all-electronic transistor action, and logic gates arising therefrom, is also known.[30,31] It is also important to note a different conceptual approach to a molecular triode based on PET, as described by Verhoeven and co-workers.[32]

**Figure 1:**
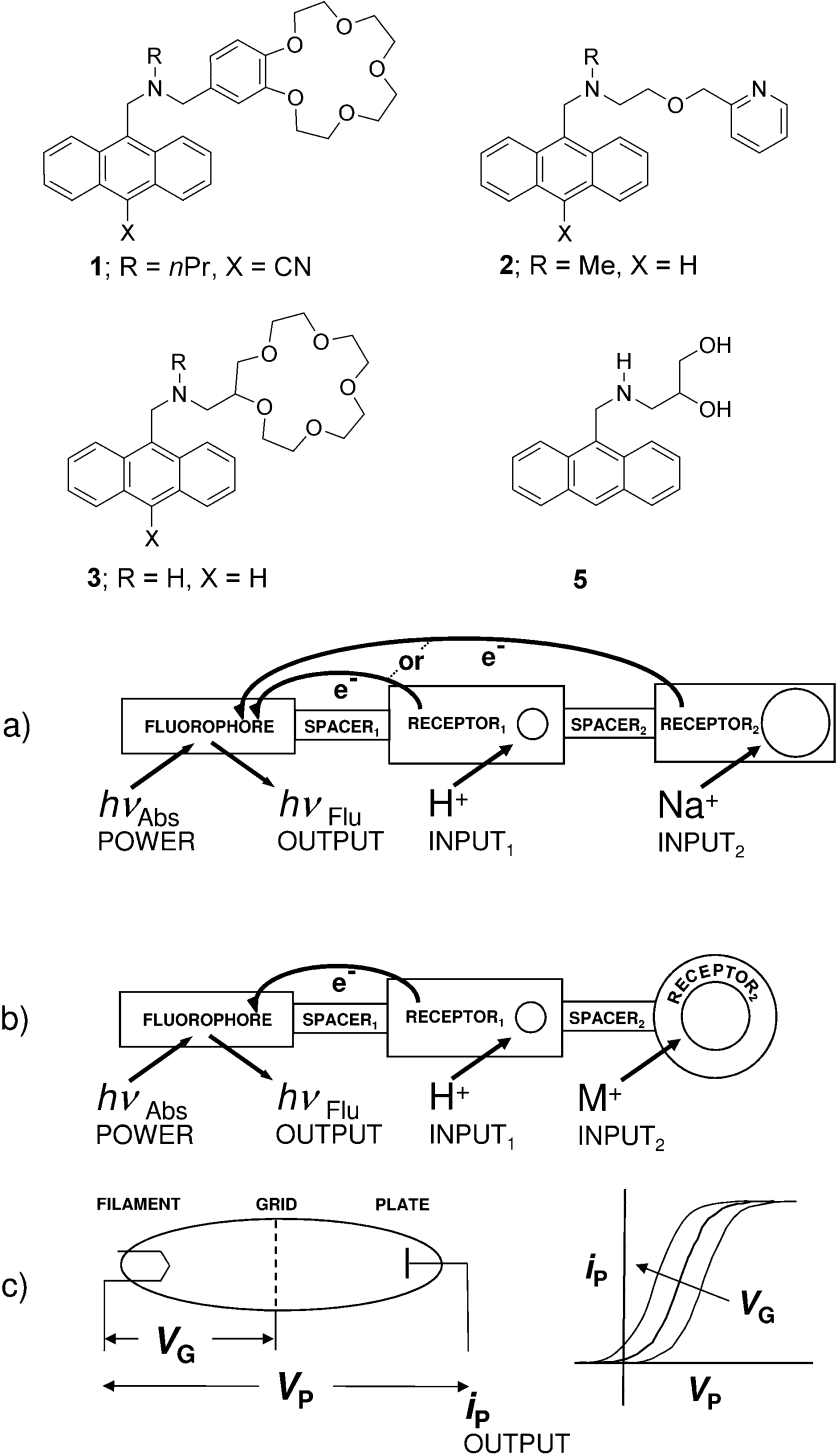
a) A fluorophore for photon transactions and two receptors for ion binding are the three crucial components of the molecular AND logic gate, where the two spacers serve as connectors. b) In a similar vein, the three crucial components of the molecular photo-ionic triode consist of a fluorophore and a principal receptor_1_ alongside an auxiliary receptor_2_. The latter endows the system with a way of tuning the input/output (I/O) characteristic curve. c) The three crucial components of the vacuum thermionic triode consist of a filament, plate, and an interspersed grid. This set-up also produces a tunable I/O characteristic.

The electronic triode[33] (the forerunner of the transistor[33]) is a fundamental switch and consists of a vacuum tube containing a hot filament undergoing thermionic emission of electrons which are collected by a plate electrode, provided the latter is at a sufficiently positive voltage. The plate current (*i*_P_) output is essentially a sigmoidal function of the plate voltage (*V*_P_) input because the electron current saturates at highly positive voltages, owing to the limited supply of electrons from the filament. The triode contains a grid electrode between the filament and the plate. The voltage of the latter (*V*_G_) sensitively controls electron traffic, that is, the *i*_P_-*V*_P_ characteristic curve is shifted along the voltage axis depending on the value of *V*_G_ (Figure 1 c). Compound **3** shows a very similar effect at the molecular level when its fluorescence emission spectrum is examined under various ionic conditions (Figure 2 a). The fluorescence quantum yield (*ϕ*_F_) output is a sigmoidal function of the pH input and the shift of the *ϕ*_F_-pH curve along the pH axis depends on the presence of another cation which lodges in the [15]crown-5 ether (Figure 2 b). Compound **3** is conveniently prepared from 9-anthraldehyde by conversion into an imine (**4**) with aminomethyl-[15]crown-5 ether, which is subsequently reduced with NaBH_4_ (see supporting information). Compound **3** is, therefore, a fluorescent PET sensor for H^+^ ions[34] with an important additional tuning element. Figure 2 b shows the data points and the fitted sigmoidal curves for each situation on employing the Henderson–Hasselbalch Equation [Eq. (1)] for the fluorescence data.[35]

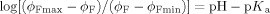
(1)

**Figure 2:**
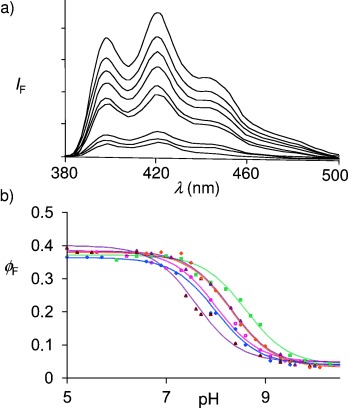
a) Fluorescence emission spectra for 10^−5^ m 3 in methanol/water (1:1, v/v) with 10^−4^ m morpholinopropylsulfonic acid in the presence of 0.3 m Me_4_NCl, when excited at 369 nm. pH adjustments were performed with Me_4_NOH and HCl. The pH values in order of decreasing fluorescence intensity are: 6.4, 7.6, 8.2, 8.5, 8.7, 8.9, 9.5, 9.8, and 10.3. It is notable that all the spectral features except the quantum yield are essentially independent of the pH value, as expected for fluorescent PET sensors containing fluorophores with ππ* excited states.[34] Similar spectra are found when Me_4_NCl is replaced by other salts (see below). b) Fluorescence quantum yield (*ϕ*_F_)-pH profiles for 3 in the presence of various chloride salts. The concentrations of monovalent cation salts and divalent cation salts were chosen to minimize ionic strength changes. The salt concentrations were chosen to allow for as much as possible of 3 to be bound to the cation through the crown ether, while respecting solubility limits. Such a choice is enabled by data tables of cation/crown ether binding constants.[46] Studies at lower salt concentrations were not conducted since those would require dissection of the *ϕ*_F_-pH profiles into metal-free and metal-bound components, with large attendant uncertainties. The cations employed are: 0.3 m Me_4_N^+^ (filled squares), 0.3 m Na^+^ (open diamonds), 0.3 m K^+^ (filled triangles), 0.1 m Ca^2+^ (filled diamonds), 0.1 m Sr^2+^ (open circles), and 0.1 m Ba^2+^ (open triangles). The full lines are calculated according to Equation (1), by employing the experimentally determined parameters p*K*_a_, *ϕ*_Fmax_, and *ϕ*_Fmin_ from Table 1.

The p*K*_a_ values determined by fluorescence spectroscopy agree with the corresponding values obtained by absorption spectroscopy, even though the latter values are only estimates because of the small absorption changes that are seen (Table 1). This is as expected for fluorescent PET sensors and switches carrying fluorophores with ππ* excited states,[25,34] since only *ϕ*_F_ is pH-dependent and since the p*K*_a_ value of the excited state is essentially identical to that of the ground state in these cases. The latter feature contributes to the operational simplicity of fluorescent PET systems compared with other sensing approaches based on fluorescence.[4] The electrostatic repulsion between the cation bound by the crown ether and the protonated amine controls the shift of the p*K*_a_ value (Table 1). Me_4_N^+^ was employed to establish the control situation where the crown ether receptor_2_ is left unbound. Sizeable Δp*K*_a_ values of 0.3, 0.3, 0.6, 0.6, and 1.0 are found with K^+^, Ca^2+^, Na^+^, Sr^2+^, and Ba^2+^, respectively. As a consequence of the good geometric fit of an Na^+^ ion into the [15]crown-5 ether cavity,[36] it produces a substantial effect, despite its single charge. A lariat action[36] from the amine side chain probably contributes to the larger dications being so effective. The tunability of the present system is already significant and sufficient for triode action, similar to the electronic version (shown schematically in Figure 1 c, right panel). Nevertheless, it should now be possible to apply the concept to other receptor pairs so that even larger tunabilities can be reached. An important issue in the development of real-life applications[7] is also addressed here—the variation of the pH value around a “normal” value (pH_normal_) is most sensitively registered by a molecular sensor if its p*K*_a_ value is matched to the pH_normal_ value. The tunability of the p*K*_a_ value of compound **3** offers a way to make this match.

**Table 1:** Acidity constants and fluorescence quantum yield data for 3 and 5.[a]

Cation	p*K*_a_ _3_	p*K*_a_ _3_[b]	*ϕ*_Fmax_ _3_	*ϕ*_Fmin_ _3_	p*K*_a_ _5_	p*K*_a_ _5_[b]	*ϕ*_Fmax_ _5_	*ϕ*_Fmin_ _5_
Me_4_N^+^	8.6	8.3	0.37	0.040	8.2	8.2	0.37	0.024
Na^+^	8.0	8.0	0.36	0.040	8.2	7.9	0.38	0.034
K^+^	8.3	8.0	0.38	0.036	8.3	8.5	0.36	0.028
Ca^2+^	8.3	8.3	0.38	0.032	8.0	–[c]	0.37	0.026
Sr^2+^	8.0	–[c]	0.38	0.048	8.0	–[c]	0.36	0.036
Ba^2+^	7.6	–[c]	0.40	0.048	8.3	–[c]	0.38	0.035

[a] Conditions as given in Figure 2. The fluorescence-based p*K*_a_ values, which were determined according to Equation (1), have uncertainties of ±0.1. The *ϕ*_F_ values have uncertainties of ±10 % and were determined by comparison with secondary standards in Ref. [35].

[b] Data estimated by analysis of small H^+^-induced changes in the UV absorption spectra according to the corresponding version of Equation (1).

[c] Spectral changes are too small to permit an estimate to be made.

We have here a rarely noted supramolecular substituent effect[37] on p*K*_a_ values, where the cation serves as the substituent. Physical organic chemistry usually deals with the effects of substituents which are covalently attached to the structure carrying the reactive site.[38] Although many cases of ion-induced p*K*_a_ shifts are available,[39–43] the concept of photo-ionic triode action is unprecedented. An elegantly tunable fluorescent PET sensor for glucose developed by James and Shinkai[44] does not correspond to photo-ionic triode action. The essential contribution of the [15]crown-5 ether module to the triode action of **3** is demonstrated by the finding that the p*K*_a_ values of control compound **5** (which is devoid of a crown ether unit) are essentially independent of the cation at 8.15±0.15 (Table 1).

To conclude, the tunable I/O characteristic of a molecular photo-ionic device which emulates thermionic triode behavior has been demonstrated for the first time by implementing a new format of fluorescent PET switches. The three-electrode philosophy of the triode is also followed in the photo-ionic system by the use of three active units within structure **3** (Figure 1 b,c). Another key aspect of triode behavior, that is, signal amplification, is well-known in other chemical systems.[45]

Dedicated to Prof. Seiji Shinkai

## References

[1] Valeur B, Berberan-Santos MN (2012). Molecular Fluorescence.

[2] Lackowicz JR (2006). Principles of Fluorescence Spectroscopy.

[3] L. Prodi, N. Zaccheroni, M. Montalti (2011). Luminescence Applied in Sensor Science.

[4] de Silva AP, Gunaratne HQN, Gunnlaugsson T, Huxley AJM, McCoy CP, Rademacher JT, Rice TE (1997). Chem. Rev.

[5] He H, Mortellaro M, Leiner MJP, Young ST, Fraatz RJ, Tusa J (2003). Anal. Chem.

[6] He H, Mortellaro M, Leiner MJP, Fraatz RJ, Tusa J (2003). J. Am. Chem. Soc.

[7] Tusa JK, He H (2005). J. Mater. Chem.

[8] He HR, Jenkins K, Lin C (2008). Anal. Chim. Acta.

[9] Yoshida K, Mori T, Watanabe S, Kawai H, Nagamura T (1999). J. Chem. Soc. Perkin Trans. 2.

[10] Kawai H, Nagamura T, Mori T, Yoshida K (1999). J. Phys. Chem. A.

[11] Batat P, Vives G, Bofinger R, Chang RW, Kauffmann B, Oda R, Jonusauskas G, McClenaghan ND (2012). Photochem. Photobiol. Sci.

[12] de Silva AP, Gunaratne HQN, Gunnlaugsson T (1998). Tetrahedron Lett.

[13] Kojima H, Nagano T (2000). Adv. Mater.

[14] Plater MJ, Greig I, Helfrich MH, Ralston SH (2001). J. Chem. Soc. Perkin Trans. 1.

[15] Domaille DW, Que EL, Chang CJ (2008). Nat. Chem. Biol.

[16] de Silva AP, James MR, McKinney BOF, Pears DA, Weir SM (2006). Nat. Mater.

[17] de Silva AP, Gunaratne HQN, McCoy CP (1993). Nature.

[18] Balzani V, Venturi M, Credi A (2008). Molecular Devices and Machines.

[19] (2012). Molecular and Supramolecular Information Processing.

[20] (2012). Biomolecular Information Processing.

[21] Szacilowski K (2012). Infochemistry.

[22] Silva APde (2013). Molecular Logic-based Computation.

[23] Pischel U, Andreasson J, Gust D, Pais VF (2013). ChemPhysChem.

[24] (2012). Molecular Switches.

[25] Bissell RA, de Silva AP, Gunaratne HQN, Lynch PLM, Maguire GEM, Sandanayake KRAS (1992). Chem. Soc. Rev.

[26] de Silva AP, Vance TP, West MES, Wright GD (2008). Org. Biomol. Chem.

[27] (2001). Electron Transfer.

[28] de Silva AP, Gunaratne HQN, McCoy CP (1996). Chem. Commun.

[29] Keirstead AE, Bridgewater JW, Terazono Y, Kodis G, Straight S, Liddell PA, Moore AL, Moore TA, Gust D (2010). J. Am. Chem. Soc.

[30] Bachtold A, Hadley P, Nakanishi T, Dekker C (2001). Science.

[31] Song H, Kim Y, Jang YH, Jeong H, Reed MA, Lee T (2009). Nature.

[32] Bakker NAC, Wiering PG, Brouwer AM, Warman JM, Verhoeven JW (1990). Mol. Cryst. Liq. Cryst.

[33] Hughes E (1990). Electrical Technology.

[34] de Silva AP, Rupasinghe RADD (1985). J. Chem. Soc. Chem. Commun.

[35] Bissell RA, Calle E, de Silva AP, de Silva SA, Gunaratne HQN, Habib-Jiwan JL, Peiris SLA, Rupasinghe RADD, Samarasinghe TKSD, Sandanayake KRAS, Soumillion J-P (1992). J. Chem. Soc. Perkin Trans. 2.

[36] Gokel GW (1991). Crown Ethers and Cryptands.

[37] Ashton PR, Fyfe MCT, Hickingbottom SK, Stoddart JF, White AJP, Williams DJ (1998). J. Chem. Soc. Perkin Trans. 2.

[38] Anslyn EV, Dougherty DA (2006). Modern Physical Organic Chemistry.

[39] Barooah N, Mohanty J, Pal H, Bhasikuttan AC (2012). J. Phys. Chem. B.

[40] Zhong ZL, Postnikova BJ, Hanes RE, Lynch VM, Anslyn EV (2005). Chem. Eur. J.

[41] Roitzsch M, Lippert B (2004). J. Am. Chem. Soc.

[42] Gerencsér L, Maroti P (2001). Biochemistry.

[43] Suga K, Ohzono T, Negishi M, Deuchi K, Morita Y (1998). Supramol. Sci.

[44] James TD, Shinkai S (1995). J. Chem. Soc. Chem. Commun.

[45] Mullis KB Angew. Chem.

[46] Izatt RM, Pawlak K, Bradshaw JS, Bruening RL (1991). Chem. Rev.

