# Supplementary material for: Modification of Fluorescent Photoinduced Electron Transfer (PET) Sensors/Switches To Produce Molecular Photo-Ionic Triode Action
Source: Angew Chem Int Ed Engl. 2014 Feb 26;53(14):3622–5. doi: 10.1002/anie.201310939 (PMC4499248; doi:10.1002/anie.201310939)
Supplement: Supplementary file 1 [file anie0053-3622-sd1.pdf]

Angewandte Chemie
Eine Zeitschrift der Gesellschaft Deutscher Chemiker

Supporting Information

© Wiley-VCH 2014

69451 Weinheim, Germany

Modification of Fluorescent Photoinduced Electron Transfer (PET) Sensors/Switches To Produce Molecular Photo-Ionic Triode Action**

*Allen J. M. Huxley, Marc Schroeder, H. Q. Nimal Gunaratne, and A. Prasanna de Silva**

anie_201310939_sm_miscellaneous_information.pdf

Supporting information

Experimental Section

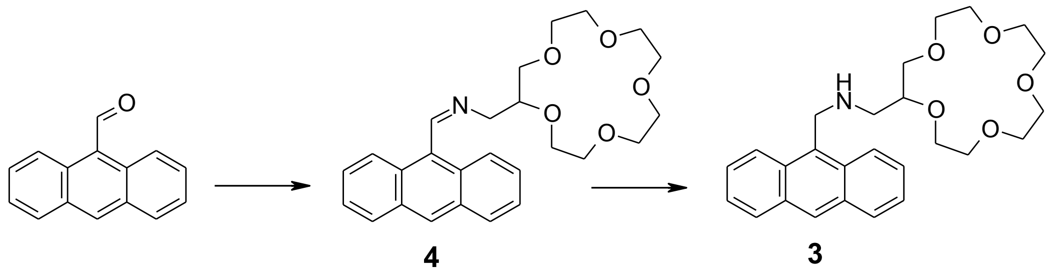

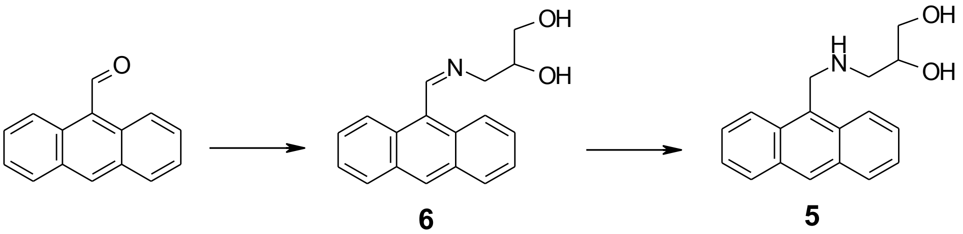

N-1-(9-anthracenyl)methylidene-N-(1,4,7,10,13-pentaoxycyclopenta decanylmethyl)amine (**4**).

Anthraldehyde (0.37 g, 1.8 mmol) was dissolved in dry chloroform and aminomethyl-15-crown-5 (0.5 g, 2 mmol) added. This was stirred under dry conditions for 4 days whereupon the chloroform was removed under reduced pressure to yield a brown oily solid (0.66 g, 84%). m/z: 437.5329, required for $C_{26}H_{31}NO_5$: 437.5332. ^{1}H NMR ($CDCl_3$) δ9.42 (s, 1H, C*H*=N), 8.52 (d, 2H, Ar*H*, J = 1.2 Hz), 8.49 (s, 1H, Ar*H*), 8.03 (d, 2H, Ar*H*, J = 1.2 Hz), 7.82 (m, 2H, Ar*H*, J = 1.2, 2.1 Hz), 7.50 (m, 2H, Ar*H*, J = 1.2, 2.1 Hz), 3.52-3.98 (m, 19H, OCH_2 and OC*H*), 2.80 (dd, 2H, NCH_2, J = 6.6, 8.7 Hz).

N-(9-anthracenylmethyl)-N-(1,4,7,10,13-pentaoxycyclopentadecanyl) amine (**3**).

Compound **4** (0.33 g, 0.76 mmol) was dissolved in dry ethanol, treated with $NaBH_4$ (0.29 g, 7.6 mmol) and refluxed for 5 hours. The mixture was cooled,

poured into CH_2Cl_2:water (1:1, v/v) and the organic layer separated. The aqueous layer was further extracted with CH_2Cl_2 (3x30 ml) and the combined organic layers evaporated to yield a brown oily solid (0.25 g, 76%). m/z: 439.5479, required for $C_{26}H_{33}NO_5$: 439.5480. ^{1}H NMR ($CDCl_3$) δ8.39 (s, 1H, Ar*H*), 8.38 (d, 2H, Ar*H*, J = 1.2 Hz), 8.03 (d, 2H, Ar*H*, J = 2.4 Hz), 7.53 (m, 2H, Ar*H*, J = 1.2, 1.8 Hz), 7.50 (m, 2H, Ar*H*, J = 1.8, 2.4 Hz), 4.74 (s, 2H, CH_2Ar), 3.78-3.83 (m, 1H, NCH_2CH), 3.62-3.70 (m, 18H, OCH_2), 2.97 (d, 2H, NCH_2CH, J = 8.1 Hz).

The control compounds **5** and **6** were prepared similarly, in yields of 86% and 81% respectively.

3-{[1-(9-anthracenyl)methylidene]amino}-1,2-propanediol (**6**).

m.p. 126-127 C. m/z: 279.1253, required for $C_{18}H_{17}NO_2$: 279.1259. ^{1}H NMR ($CDCl_3$) δ9.46 (s, 1H, CH=N), 8.51 (s, 1H, ArH), 8.47 (d, 2H, ArH, J = 0.6 Hz), 8.01 (d, 2H, ArH, J = 1.5 Hz), 7.49-7.57 (m, 4H, Ar*H*, J = 0.6, 1.5, 5.1 Hz), 4.17-4.20 (m, 1H, NCH_2CH, J = 0.6, 7.5 Hz), 4.07 (d, 2H, NCH_2, J = 0.6 Hz), 3.87 (d, 2H, NCH_2CHCH_2, J = 7.5 Hz), 2.88 (br, 1H, CHO*H*), 2.65 (br, 1H, CH_2O*H*).

3-[(9-anthracenyl)methylamino]-1,2-propanediol (**5**).

m.p. 119-120 C. m/z: 281.1408, required for $C_{18}H_{19}NO_2$: 281.1416. ^{1}H NMR ($CDCl_3$) δ8.34 (s, 1H, Ar*H*), 8.22 (d, 2H, Ar*H*, J = 7.0 Hz), 7.94 (d, 2H, Ar*H*, J = 8.1 Hz), 7.44 (m, 4H, Ar*H*, J = 6.9, 8.1 Hz), 4.64 (m, 2H, $ArCH_2$), 3.70 (br, 1H, N*H*), 3.56 (dd, 2H, NCH_2CH, J = 3.3, 8.1 Hz), 3.45 (m, 1H, NCH_2CH), 2.75-2.93 (m, 4H, NCH_2CHCH_2 and CHO*H* and CH_2O*H*).
